# Pap Smear miR-92a-5p and miR-155-5p as Potential Diagnostic Biomarkers of Squamous Intraepithelial Cervical Cancer

**DOI:** 10.31557/APJCP.2021.22.4.1271

**Published:** 2021-04

**Authors:** Tahereh Azimi, Mahdi Paryan, Mahdieh Mondanizadeh, Hossian Sarmadian, Ashraf Zamani

**Affiliations:** 1 *Department of Biotechnology and Molecular Medicine, Arak University of Medical Sciences, Arak, Iran. *; 2 *Department of Research and Development, Production and Research Complex, Pasteur Institute of Iran, Tehran, Iran. *; 3 *Molecular and Medicine Research Center, Arak University of Medical Sciences, Arak, Iran. *; 4 *Department of Infectious Diseases Research Center, School of Medicine, Arak University of Medical Sciences, Arak, Iran. *; 5 *Department of Obstetrics and Gynecology, School of Medicine, Arak University of Medical Sciences, Arak, Iran. *

**Keywords:** LSIL, HSIL, miRNAs, biomarker, HPV E6/E7

## Abstract

**Background:**

one of the female-specific diseases with a high incidence and mortality is cervical cancer. The main cause of cervical cancer is infection with Human papilloma virus (HPV). Low-grade squamous intraepithelial lesions (LSIL) and High-grade squamous intraepithelial lesions (HSIL) usually is caused by an HPV infection. Considering the role of microRNAs (miRNAs) as diagnostic biomarkers for a variety of cancers, the aim of this study was to determine miR-92a-5p and miR-155-5p expression levels in LSIL and HSIL Pap Smear samples.

**Methods:**

After initial bioinformatic studies, A total of 75 samples (25 samples of patients with LSIL, 25 patients with HSIL and 25 healthy individuals) were subjected to RNA extraction and cDNA synthesis. The expressions levels of confirmed miRNAs in samples of patients with LSIL, HSIL and healthy individuals were evaluated by Real time PCR analysis. To demonstration the role of predicted miRNAs as novel biomarkers in diagnosis of LSIL and HSIL, ROC curve analysis was done.

**Results:**

Bioinformatics results showed that miR-92a-5p and miR-155-5p target the HPV E6 and E7 genes. The expression levels of these miRNAs were strikingly higher in Pap smear of patients with LSIL than in the healthy individuals (35.36, P = 0.001) (62.23, P = 0.001). Similarity, expression levels of miR-92a-5p and miR-155-5p were amazingly higher in patients with HSIL than in the healthy individuals (33.62, P= 0.001) (69.07, P= 0.001). Although, the levels of miR-92a-5p (0.95, P = 0. 85) and miR-155-5p (1.11, P = 0.84) exhibited no statistical differences between patients with LSIL and HSIL. Also, ROC curve analyses verified that miR-92a-5p and miR-155-5p are specific and sensitive and may serve as new biomarkers for the early detection of cervical cancer.

**Conclusion:**

These data suggest miR-92a-5p and miR-155-5p, which are upregulated in LSIL and HSIL, can be consider as predictive biomarkers for the prognosis of cervical cancer patients.

## Introduction

Cervical cancer (CC) is categorized as the 4^th^ most common malignancy in women worldwide (Bray et al., 2018; Canfell et al., 2020)and nearly 90% of deaths from cervical cancer raised in low- and middle-income countries (Tsikouras et al., 2016). The annual incidence of this malignancy is more than 550,000 cases and more than 275,000 deaths in women (Arbyn et al., 2011). High risk human papillomavirus (HR-HPV) infection is distinguished as the most important etiologic factor in cervical cancer (Tian et al., 2014; Bray et al., 2018) Which causes more than 99% of this cancer cases (Nguyen et al., 2014). The E6 and E7 proteins in the HPV genome are known as viral oncogenes (Pinidis et al., 2016) , as well as Long-term over-expression of HPV E6 and E7 oncogenes have a crucial role in the development of cervical cancer(Bano et al., 2018). These proteins induce cell neoplastic transformation because of genetic and epigenetic instability (Park et al., 2017). In additional tumor suppressor genes p53 and Rb were inactivated by HPV E6 and E7, respectively (Banno et al., 2014; Park et al., 2017). In recent years, the techniques and methods available for screening and diagnosis of cervical cancer in the early stages are invasive and inappropriate. Therefore, there is a need for biomarkers that have the ability to diagnose this cancer in the early stages, with a high sensitivity and specificity. On the other hand, microRNAs effect on epigenetic instability. They are small non-coding RNA molecules with a length of 19 to 25 nucleotides and have a significant role in post-transcription and epigenetic regulation via binding to 3`-UTR of target mRNAs. miRNAs are associated in pathological and physiological events (Ma et al., 2014) .Their dysregulation is involved in different types of human malignancies such as colon cancer, breast cancer, lung cancer as tumor suppressor genes or oncogenes(Tian et al., 2014; Park et al., 2017). Dysregulated expression of miRNAs in cancers implies that the identification of miRNAs and their target molecules is essential for understanding the pathways leading to cancer (Gocze et al., 2015). Moreover, these evidences indicate they have potential application as biomarkers for cervical cancer screening. In previous studies, some miRNAs were introduced for screening of cervical cancer with different methods. Xia et al. conducted a study to examined the association between HPV infection and miRNA expression using Microarray in culturing cells transfected with E6 and E7 genes (Zheng et al., 2019). Honegger et al. investigated the expression of intracellular and exosomal miRNAs by altering the expression of E6 and E7 genes using deep sequencing and RT-qPCR (Honegger et al., 2015). Louise et al. considered the expression of miRNAs in CIN and tissue samples from 120 individuals using a microarray technique in order to find biomarkers for HPV infection or progression (Wang et al., 2014).

Therefore, the purpose of this study was to select the most important miRNAs targeting E6 and E7 oncogenes in the HPV virus with the help of bioinformatics studies, and then compare the expression level of selected miRNA in the LSIL, HSIL and healthy individuals’ samples.

## Materials and Methods

*Clinical samples*

A total of 25 LSIL Pap smears (Pap test) samples, 25 HSIL Pap Smear samples and 25 healthy individuals with no current or previous malignancy were collected from Imam Reza clinic and valiasr hospital (Arak, Iran). The technology of liquid-based cytology (LBC) was applied for the Pap smear. All Cases were reviewed by two pathologists and Cytological results were categorized according to Bethesda classification (Solomon et al., 2002) . None of the patients had chemotherapy, radiotherapy or other treatment history or other inflammatory diseases moreover in order to eliminate the disruptive effect of smoking, all samples were either not smokers or quit. (Table 1). The study approved in Arak University of medical science ethical committee and The Ethic Approval Code is IR.ARAKMU.REC.1396.296.

*miRNAs selection*

Previous studies identify that several miRNAs, including miR-34a, miR-92a-5p, miR-155-5p and miR-195-3p, are closely associated with the prognosis cervical cancer and Pre-malignant lesions (Chen et al., 2017a; Song et al., 2017; Su et al., 2017; Li et al., 2019). Furthermore, using the NCBI database, the E6 and E7 genes sequences were retrieved for both human papillomavirus types 16 and 18. Then, with help of miRBase (www.mirbase.org) and RNA22 (http://cm.jefferson.edu/rna22/Interactive/) bioinformatics softwares, binding of selected the miRNAs to E6 and E7 genes was confirmed.

*Primer design*

The sequences of the miRNAs were acquired from miRBase database. Reverse transcription-specific stem-loop primers and gene specific primers were designed using the AlleleID7 and GeneRunner software. The expression of miR-103 was used as endogenous reference gene for normalization of predicted miRNAs. The specificity of the designed primers was determined using the nucleotide BLAST on NCBI. The sequences of Stem-loop gene-specific primers, forward and the universal reverse primers are summarized in Table 2.

*miRNA extraction and Reverse transcription*

microRNAs were extracted from Pap smears samples using RNX-Plus reagent (SinaClon, Iran) according to the manufacturer`s instructions. All the procedures were carried out under RNase-free conditions. RNA concentrations were measured using a NanoDrop and isolated microRNAs was stored at −70°C until used. Complementary DNA (cDNA) was synthesized using the mixture of M-MLV enzyme (Vivantis, Malaysia) according to manufacturer’s instructions. Briefly, 1 μg of isolated microRNAs was used for cDNA synthesis. The reverse transcriptase (RT) reaction mixture contained the combination of M-MLV enzyme, 1x RT-enzyme buffer, 400 μM dNTP, and 1μM of specific stem-loop RT primers and adjusted the total reaction volume to 15 μL with nuclease free water. The cDNA synthesis reaction was performed as follows: incubated at 70^o^C for 5 min and then followed by 37°C for 60 min, in a thermal cycler (Eppendorf, Germany). Eventually, the obtained cDNAs were stored at -20˚C prior to RT-qPCR analyses.

*Quantitative Real-time PCR*

All RT-qPCR analyses were performed in a Light Cycler 96 instrument (Roche, Germany) in triplicates. The reaction mixture contained 7.5 µL of 1x SYBR Green PremixExRaq II (Yekta Tajhiz Azma, Iran), 1.5 µL cDNA, and 0.5 µM of each forward and reverse primers, and 5µL RNase-free water to adjust the reaction volume to 15 µL. PCR cycling conditions were as follows: 95ºC for 3 min, 40 cycles of 95ºC for 10 seconds, 55 ºC for 15 seconds and 72ºC for 20 seconds. Melting curve analysis was performed after amplifications from 60°C to 96°C with a ramp rate of 0.2°C/second and continuous fluorescence acquisition. The relative expression was computed by the comparative Cq method using the relative expression software tool (REST 2009) (Pfaffl et al., 2002).

*Statistical methods*

Analyze of this RT-qPCR data was performed using REST 2009 software and the data in graphs are expressed as the mean ± SE. Receiver operating characteristic (ROC) curves analysis were created to assess diagnostic value of each miRNA, and the area under the ROC curve (AUC) was calculated to measure discriminatory capacity. The best sensitivity/specificity pair was selected based on the maximum likelihood ratio. All statistical analyses were calculated using SPSS software (version 16; SSPS Inc., 184 Chicago). P-values of <0.05 was considered statistically significance.

## Results

*Confirmed of the selected miRNAs*

Findings from bioinformatics studies confirmed binding miR-34a, miR-92a, miR-155 and miR-195 to E6 and E7 mRNAs. These results have computed and are shown in Table 3. If there are no results shown in RNA22 software, it means your chosen parameters yielded no results.

*HPV E6 and E7 transcripts targeting miRNAs analysis using RT-qPCR*

**Figure 1:**
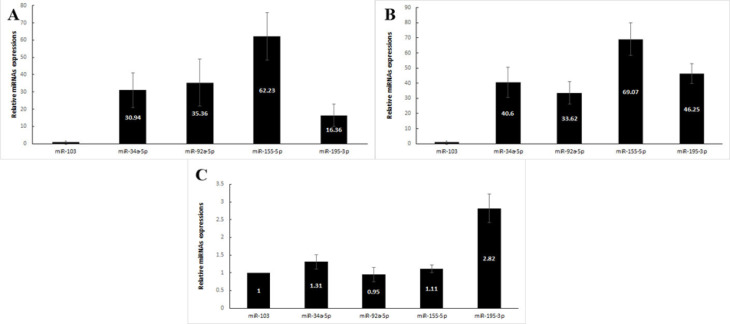
(A) Comparison of differential expression levels of miR-34a-5p, miR-92a-5p, miR-155-5p and miR-195-3p between patients with LSIL and healthy individuals. B) patients with HSIL and healthy individuals. (C) patients with HSIL and LSIL. Error bars indicate the standard error of the mean

Expression levels of four candidate miRNAs were investigated in 25 LSIL, 25 HSIL Pap smears samples and 25 healthy controls. As summarized in Figure 1A, the RT-qPCR analysis showed that the relative expression levels of miR-34a-5p, miR-92a-5p, miR-155-5p and miR-195-3p were significantly higher in patients with LSIL than in healthy controls (30.94, P = 0.001) (35.36, P = 0.001) (62.23, P = 0.001) and (16.36, P = 0.001), respectively.

Similarly, the expression levels were 40.60 folds (P = 0.001) for miR-34a-5p, 33.62 (P = 0.001) for miR-92a-5p, 69.07 (P = 0.001) for miR-155-5p and 46.25 (P = 0.001) for miR-195-3p in patients with HSIL compared whit healthy controls, respectively (Figure 1B). 

The expression levels of the four miRNAs were also compared between patients with HSIL and LSIL. The Pap smear level of miR-195-3p (2.82, P = 0.01) was significantly higher in patients with HSIL compared whit LSIL, whereas the levels of miR-34a-5p (1.31, P = 0.72), miR-92a-5p (0.95, P = 0. 85) and miR-155-5p (1.11, P = 0.84) exhibited no statistical differences between the two groups (Figure 1C).

*Capability of miR-92 and miR-155 to function as diagnostic biomarkers for LSIL and HSIL prognosis*

We plotted ROC curves to determine quantification values of miR-34a-5p, miR-92a-5p, miR-155-5p, and miR-195-3p, in an attempt to differentiate LSIL and HSIL patients from healthy individuals. The cutoff levels were elected as the point with the maximum sum of sensitivity and specificity on the ROC curves using the Youden`s index ([sensitivity + specificity] -1) (Khansarinejad et al., 2015).

**Figure 2:**
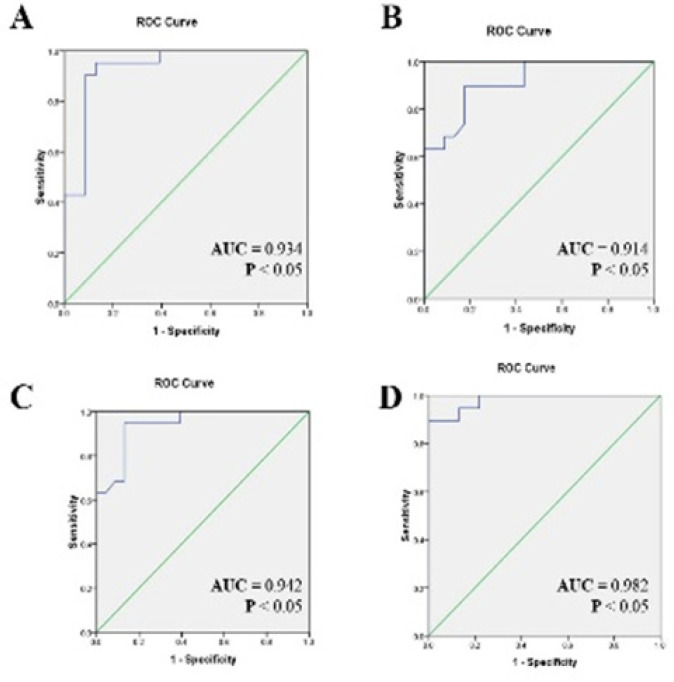
Receiver Operating Characteristic (ROC) Curve Analysis was Performed to Determine the Sensitivity and Specificity of (A) the miR-92a-5p and (B) miR-155-5p expression levels between the patients with LSIL and the healthy individuals (C) The miR-92a-5p and (D) miR-155-5p expression levels between the patients with HSIL and the healthy individuals, using area under the ROC curve (AUC) analysis

Comparisons of the miRNA Pap Smear levels between LSIL patients and healthy individuals showed that at the cutoff level of 1.93, miR-92a-5p had 95% sensitivity and 87% specificity with an area under curve (AUC) of 0.934. At the cutoff point of 2.1, miR-155-5p exhibited 89%sensitivity and 83% specificity, with an AUC of 0.914 (Figure 2A and 2B, Table 4).

On the other hand, in comparison between HSIL patients and healthy individuals, at the cutoff point of 2.08 the sensitivity, specificity, and AUC for miR-92a-5p are 94%, 87% and 0.942, respectively. The cutoff point of 3.17 of miR-155 had a sensitivity of 95%, specificity of 83%, and AUC of 0.982. (Figure 2C and 2D, Table 4).

Regarding the over expression of miR-34a-5p and miR195-3p in LSIL and HSIL patients Compared with healthy individuals, rock curve was plotted only for miR-92a-5p and miR-155-5p. On the other hand, because of the expression levels of miR-34-5p (1.31, P = 0.72), miR-92a-5p (0.95, P = 0.85), and miR-155-5p (1.11, P = 0.84) exhibited no statistical differences between patients with LSIL and patients with HSIL, ROC curve analyses were also performed to assess the possibility of using pap smear levels of miR-92a-5p and miR-155-3p as diagnostic biomarkers for detecting patients with LSIL and HSIL from healthy individuals.

**Table 1:** Sample Information in LSIL, HSIL and Healthy Individuals

Available	Total	Histology			Age		Smoking status	
		LSIL	HSIL	Normal	<50 years	≥50 years	Never	quit
Patients Number (%)	50 (66.6%)	25 (33.3%)	25 (33.3%)		32(64%)	18(36%)	41(82%)	9(18%)
Healthy controls Number (%)	25 (33.3%)			25 (33.3%)	17(68%)	8(32%)	18(72%)	7(28%)

**Table 2:** Primers Used for Reverse-Transcription and RT-qPCR Assay of the Target miRNAs

Target	Primer sequences (5`-3`)
miR-34a	S^a^: 5`-GGTCGTATGCAGAGCAGGGTCCGAGGTATCCATCGCACGCATCGCTCTGCATACGACCACAACCA-3`
	F^b^: 5`-GGGTTGGCAGTGTCTTAGC-3`
miR-92a-5p	S: 5`-GGTCGTATGCAGAGCAGGGTCCGAGGTATCCATCGCACGCATCGCTCTGCATACGACCAGCA-3`
	F: 5`-AGGTTGGGATCGGTTGC-3`
miR-155-5p	S: 5`-CTGTTAATGCTAATCGTGATAGGGGTTTTTGCCTCCAACTGACTCCTACATATTAGCATTAACAG-3`
	F: 5`-TTAATGCTAAUCGTGATAGGGGTT-3`
miR-195-3p	S: 5`-GGTCGTATGCAGAGCAGGGTCCGAGGTATCCATCGCACGCATCGCTCTGCATACGACCGGAG-3`
	F: 5`-CACCAATATTGGCTGTGCTG-3`
miR-103	S: 5`-GTCGTATCGAGAGCAGGGTCCGAGGTATTCGCACTCGATACGACCAAGGCA-3`
	F: 5`-GCTTCTTTACAGTGCTGCC-3`
Common Reverse	5`-AGAGCAGGGTCCGAGGT-3`

^a^, Stem-loop gene-specific reverse-transcriptase primers; ^b^, Specific forward primers for RT-qPCR amplification

**Table 3:** The Result of Confirm Binding of miRNAs to HPV E6 and E7 Transcripts

Gene name	miRNA	Folding energy (in-Kcal/mol)	P-Value*	ID Number
*E6 (HPV16)*	has-miR-155-5p	-12.7	4.95E-02	MIMAT0000646
*E6 (HPV18)*	has-miR-195-3p	-13	3.99E-02	MIMAT0004615
	has-miR-34a-5p	-14.4	2.73E-01	MIMAT0000255
*E7 (HPV16)*	has-miR-92a-5p	-17.7	7.51E-02	MIMAT0004507
	has-miR-195-3p	-13.4	2.24E-01	MIMAT0004615
*E7 (HPV18)*	has-miR-34a-5p	-16.5	5.33E-02	MIMAT0000255

*The p-value represents the likelihood that the target site loci are random. That is, a lower p-value represents a greater chance

**Table 4:** Comparison of the miRNA’s Diagnostic Efficiency between the LSIL and HSIL Patients vs. the Healthy Individuals

miRNA	AUC*	95% CI**	sensitivity	specificity	cutoff level	Y-index***
miR-92a-5p (LSIL)	0.934	0.858-1.01	95%	87%	1.93	0.82
miR-155-5p (LSIL)	0.914	0.832-0.997	89%	83%	2.1	0.72
miR-92a-5p (HSIL)	0.942	0.876-1.007	94%	87%	2.08	0.82
miR-155-5p (HSIL)	0.982	0.832-0.997	95%	83%	3.17	0.77

* AUC, an area under the ROC curve; ** CI 95%, 95% confidential interval; ***, The Y-index was used to judge the most appropriate critical point. It was calculated as follow (1- [sensitivity+ specifity]).

## Discussion

Cervical cancer is one of the most common malignancies worldwide that can be treated by screening and early detection (Denny, 2012). Today, different methods such as Pap Smear, colposcopy and biopsy are used to diagnose precancerous cervical lesions (Livingston and Papagiannakis, 2016). The major problem with these methods is their low specificity and sensitivity and their aggressiveness. Therefore, this cancer remains one of the most serious health problems in the world . It is now known that miRNAs are closely linked to various diseases, including cancer, because they are involved in all biological processes including cell growth and differentiation, cell cycle regulation, stress response, and apoptosis (Esquela-Kerscher and Slack, 2006). Previous extensive studies have suggested a link between the expression of miRNAs with the onset and progression of various cancers. Therefore, altering the expression profile of miRNAs can be used as possible biomarkers of cancer development (Hu et al., 2010). Some previous studies indicate alterations in the expression of some cellular miRNAs by the E6 and E7 viral oncogenes (Honegger et al., 2015). Currently, most studies on cancer and miRNAs have been performed on tissue specimens, although these studies have greatly helped to raise the level of scientific research related to cancer, but the use of tissue for cancer detection in turn it is an invasive method. Due to its high stability and relatively easy detection miRNA compared to mRNA, they could be applied as good candidates for diagnostic in oncology. Therefore, to the best of our knowledge, there was no published report about the application of Pap smear specimens for evaluating the expression of miR-34a-5p, miR-92a-5p, miR-92a-5p, and miR-195-3p using RT-qPCR in patients with different degrees of pre-malignant lesions. The bioinformatics predictions offered here indicated that miR-34a-5p, miR-92a-5p, miR-92a-5p, and miR-195-3p could target the transcript of the E6 and E7 genes. In addition, these chosen miRNAs from literature were confirmed using RNA22 software. These chosen miRNAs frequently dysregulated in many cancers and they can act as a tumor suppressor or an oncogene depending on cellular type (Rusek et al., 2015). miR-34a-5p, targets E6 and E7 (HPV18), is identified as a crucial regulator of tumor suppression. Theoretically, P53 controls expression of miR-34a transcriptionally that disrupted in many cancers. So activated p53 induces the transcription of mir-34a which is involved in cellular transformation and carcinogenesis by targeting of several molecules. miR-34a-5p is frequently downregulated in both cancer tissues and cervical cell lines, compared with normal tissues (Zhu et al., 2018). Although we have found increase of miR-34a-5p expression in LSIL and HSIL patients rather normal individual. Comparison of expression levels between HSIL and LSIL patients, revealed no significant difference in expression levels for miR-34a-5p. Our work shows controversial results regarding miR-34a, the increase of miR-34a expression in HPV pre-infectious cells is probably explained by the activation of cellular repair mechanisms after viral infection that would activate p53 pathways and therefore, induce miR-34a expression (Ribeiro et al., 2015). Hence, it is important to perform more studies on miR-34a-5p levels in HPV infection and cervical lesions/cancer and to clarify whether there are other pathways besides the p53-associated pathways, which are able to activate miR-34a expression in HPV-associated lesions. miR-92a, a member of the miR-17-92 family, has been described to play an oncogenic role in cervical cancer (Su et al., 2017). Several studies have identified increase in levels of miR-92 in cervical tissues and cell lines (Wang et al., 2014), but none of these studies evaluated the pap smear levels of this molecule as a potential biomarker in cervical pre-malignant lesions. miR-92a-5p is thought to induce its effects by targeting essential molecules in the cell cycle, including Retinoblastoma (RB), Dickkopfrelated protein 3 (DKK3) and FBXW7 mRNA and then, promotes cell proliferation and invasion in cervical cancer (Zhou et al., 2015; Chen et al., 2017b; Yu et al., 2019). The bioinformatics predictions in the current study indicate that miR-92a-5p might directly target the E7 mRNA of the HPV16. Therefore, cervical cancer progression may be promoted by the expression of it in cancer tissues through the expression of E7 protein. Previous investigations suggested that miR-155 is closely associated with the prognosis of patients with cervical cancer. it was markedly overexpressed in cervical cancer tissues and cell line when compared to normal tissues and miR-155 increases the proliferation of cervical carcinoma cell via targeting its targets gene liver kinase B1 and TP53INP (Xu et al., 2019). In addition, miR-155-5p is dysregulated in several types of malignancies, including breast cancer, colon carcinoma, hepatocellular carcinoma and gastric cancer (Li et al., 2019). Considering the role of mi-RNA in induction of gene expression by activating target gene promoter, miRNAs Transcriptional Gene Activation (TGA) , overexpression of miR92-5p and miR-155-5p despite targeting E6 and E7 genes can be justified in this study (Janowski et al., 2007; Place et al., 2008; Ramassone et al., 2018). However, in the case of overexpression of these two miRNAs, our results are in line with previous studies (Su et al., 2017; Li et al., 2019). In the study of Philp et al and SONG et al, it was shown that miR195 could act as a tumor suppressor gene in cervical cancer, therefore, its expression in cervical cancer tissues was downregulated compared to normal tissues (Song et al., 2017; Li et al., 2018; Yang et al., 2019). These researchers explored the negative relationship among miR-195-5p whit MMP14 (Matrix metalloproteinases 14) and HDGF (hepatomaderived growth factor). It is reported that MMP14 act as a tumor promoter and HDGF is involved in the regulation of cell apoptosis, angiogenesis, invasion and metastasis and they were targeted by miR-195-5p (Song et al., 2017; Li et al., 2018) . The results are incompatible with our findings in our study, expression of miR195-3p was increased. Moreover, our bioinformatic analysis was shown miR-195-3p, targets E6 and E7 (HPV18). For the reasons mentioned in Section 3-3, the Roc curve was drawn only for to evaluate the likelihood of using Pap smear levels of miR-92a-5p and miR-155-3p as diagnostic biomarkers for distinguishing patients with LSIL and HSIL from healthy individuals. In the first Roc calculation, the microRNA levels were compared between LSIL patients and healthy individuals. The values for miR-92a-5p and miR-155-5p showed the almost equal of AUC, sensitivity, specificity, and cutoff point (Figure 2). Therefore, the expression of these two molecules could accurately distinguish healthy individuals from patients with LSIL, with an excellent sensitivity and specificity. The second ROC analysis compared the Pap smear levels of the miRNAs between the HSIL and healthy individuals. The values for miR-92a-5p and miR-155-5p presented again the almost similar sensitivity, specificity, and AUC, and, cutoff of point. Our study has some limitations such as the following: the limited number of samples and lack of ICC (invasive cervical carcinoma.) group.

In conclusion, the current study offered that Pap smear miRNAs could be promising novel biomarkers for the diagnosis of cervical cancer. Additionally, it is the first report related to a biological effect for miR-92a-5p and miR-155-5p, in order to prognose of cervical cancer. Moreover, our results offer that over expression of miR-92a-5p and miR-155-5p in the Pap smear may have roles in LSIL and HSIL emergence or progression. 

*Abbreviations*

HPV: Human papilloma virus; LSIL: Low-grade squamous intraepithelial lesions; HSIL: High-grade squamous intraepithelial lesions; miRNAs: microRNAs; HR-HPV: High risk human papillomavirus; LBC: liquid-based cytology; cDNA: Complementary DNA; RT-PCR: Reverse Transcriptase-PCR; ROC: Receiver operating characteristic; AUC: Area Under the ROC Curve; CI 95%: 95% confidential interval; RB: Retinoblastoma; DKK3: Dickkopfrelated protein 3; MMP14: Matrix metalloproteinases 14; HDGF: Hepatomaderived growth factor; TGA: Transcriptional Gene Activation.

## Author Contribution Statement

All authors made substantial contributions to conception and design, acquisition of data, or analysis and interpretation of data; took part in drafting the article or revising it critically for important intellectual content; gave final approval of the version to be published; and agree to be accountable for all aspects of the work.

## Data Availability

All relevant materials are described in the manuscript. Additional data sets supporting the conclusions of this article are available at request from the corresponding author.
